# COVID-19 and Carcinogenesis: Exploring the Hidden Links

**DOI:** 10.7759/cureus.68303

**Published:** 2024-08-31

**Authors:** Özgür Tanrıverdi, Ali Alkan, Turan Karaoglu, Sait Kitaplı, Aysegul Yildiz

**Affiliations:** 1 Medical Oncology, Muğla Sıtkı Koçman University Faculty of Medicine, Muğla, TUR; 2 Primary Health Care, Bodrum State Hospital, Muğla, TUR; 3 Molecular Biology and Genetics, Muğla Sıtkı Koçman University Faculty of Medicine, Muğla, TUR

**Keywords:** epigenetic changes, inflammatory cytokines, covid-19 outbreak, cancer epidemiology, carcinogenesis, cancer cell biology

## Abstract

The COVID-19 pandemic caused by the SARS-CoV-2 virus has been studied predominantly in terms of its immediate respiratory and systemic effects. However, emerging evidence suggests possible long-term effects, including its role in carcinogenesis. This comprehensive review explores the complex relationship between COVID-19 and cancer development, focusing on immune dysregulation, chronic inflammation, genetic and epigenetic alterations, and the impact of therapeutic interventions. We also focused on the molecular mechanisms by which SARS-CoV-2 may facilitate cancer progression, including the roles of angiotensin-converting enzyme 2 (ACE2), transmembrane serine protease 2 (TMPRSS2), and FURIN. Additionally, we examined the possible carcinogenic effects of long-term COVID-19 treatments and the interaction between co-infections and cancer risk. Our findings highlight the need for increased cancer surveillance in COVID-19 survivors. In the post-COVID-19 period, it can be thought that inflammation associated with excessive cytokine release, especially interleukin-6, genetic and epigenetic changes, and co-infections with oncogenic viruses such as Epstein-Barr virus or human papillomavirus may be effective in the development and progression of cancer. Further research is needed to explain the mechanisms underlying this relationship.

## Introduction and background

In December 2019, a disease that rapidly spread and caused acute respiratory stress syndrome was identified after a group of patients were clustered as atypical viral pneumonia in the Wuhan Province of the People's Republic of China [1-3]. After the rapid microbiological and molecular examinations, it was found that the factor that caused this clinical picture, which has a high rate of admission to the intensive care unit (ICU), intubation, and mortality, was a new coronavirus (nCoV) [1,2]. It was determined after virological investigations that this virus was a member of the beta-coronavirus family, including the severe acute respiratory syndrome coronavirus (SARS-CoV), which caused deaths in 2002. This caused the new virus to be named SARS-CoV-2 by the World Health Organization (WHO), which spread rapidly all over the world. The WHO named the disease by this virus, which caused rapid pulmonary destruction, the coronavirus disease 2019 (COVID-19) [2,4].

Although CoVs, which cause mild respiratory symptoms, were not seen as serious viruses before 2002, SARS-CoV and the Middle East Respiratory Syndrome (MERS)-CoV epidemics have been identified by researchers as an interesting family of viruses after 2002 [1,5]. However, it was concluded that after the COVID-19 pandemic caused by SARS-CoV-2, in which the whole world was heavily affected and caught off guard, many more features related to CoVs were unknown earlier [3,4].

Beyond the acute respiratory syndrome and systemic complications, there is growing concern about the long-term effects of COVID-19, including its potential role in carcinogenesis [5-10]. Carcinogenesis, the process by which normal cells transform into cancer cells, involves a complex interplay of genetic, epigenetic, and environmental factors. The question of whether carcinogenesis can be triggered in asymptomatic COVID-19 carriers or patients who have recovered from COVID-19 can be considered a hypothesis that is being discussed as a gray zone. The basis of this hypothesis is based on the fact that COVID-19 reduces the level of angiotensin-converting enzyme 2 (ACE2) in the severe disease phase (since it is exposed to proteolysis by the virus) and initiates a series of cytokine reactions with an increase in angiotensin II (AGTII) levels [6-10]. With the inflammatory response that occurs after the increase in AGTII, a serious pulmonary destruction process continues in which the immune system and inflammation as well as thrombotic events develop. However, there is no clear information about the inflammatory imbalance caused by AGTII levels in COVID-19 survivors or asymptomatic carriers and the integration process of the host cell with the virus genome [6-10]. In addition, it has been shown that some proteins belonging to SARS-CoV viruses can modulate extrinsic and intrinsic apoptosis pathways in the host cell at many stages due to their pro-apoptosis and anti-apoptosis properties. Data on the consequences of viral genomic integration in recovered patients, vaccinated individuals, or asymptomatic carriers are not yet available.

This comprehensive review aims to investigate possible links between COVID-19 and carcinogenesis by examining current literature on molecular and cellular mechanisms that may facilitate cancer development in COVID-19 patients, inspired by the molecular virological properties of SARS-CoV2.

## Review

A comprehensive literature search was conducted using databases such as PubMed, Scopus, and Web of Science. The search terms included “COVID-19,” “SARS-CoV-2,” “Carcinogenesis,” “Cancer,” “Oncogenesis,” and “Long-term effects.” The search was limited to studies published between January 2020 and June 2024. Additional studies were identified through manual searches of reference lists from relevant articles. The prominent findings of the review of these articles regarding the relationship between COVID-19 and carcinogenesis are systematically presented below.

Mechanisms of COVID-19-associated immune dysregulation

The relationship between COVID-19 and the immune system is a complex and multifaceted issue that warrants extensive investigation. COVID-19 induces a significant immune response, often characterized by a cytokine storm, which can lead to chronic inflammation. Chronic inflammation is a well-known risk factor for cancer development. Studies have shown that prolonged inflammatory responses can result in DNA damage, promote cellular proliferation, and inhibit apoptosis, thereby facilitating carcinogenesis [6-10].

COVID-19 causes a disruption in the immune system’s normal functioning, leading to an overproduction of pro-inflammatory cytokines. This immune dysregulation can result in an environment conducive to tumor growth. The prolonged presence of inflammatory cytokines such as interleukin (IL)-6, tumor necrosis factor (TNF)-α, and IL-1β can cause oxidative stress and DNA damage, key factors in carcinogenesis [6-10]. The implications of these findings are profound, given the widespread and ongoing nature of the COVID-19 pandemic. The inflammatory response triggered by SARS-CoV-2 not only affects the acute phase of the disease but also has potential long-term effects on survivors’ health, particularly in terms of cancer risk. The cytokine storm associated with severe COVID-19 can lead to significant immune dysregulation, creating an environment that fosters carcinogenesis. Additional studies support these findings. For instance, Yin et al. reported that COVID-19 patients exhibited elevated levels of pro-inflammatory cytokines even months after recovery, suggesting a prolonged inflammatory state [11]. Their study found a 35% increase in IL-6 and a 28% increase in TNF-α levels three months post-recovery. Similarly, Zhang et al. observed that long-term COVID-19 survivors had higher levels of oxidative stress markers, which correlated with increased cancer risk factors [12]. Their longitudinal study showed a 40% increase in oxidative stress markers and a 22% increase in DNA damage markers in COVID-19 survivors over a year.

Furthermore, emerging evidence suggests that the impact of COVID-19 on the immune system might be even more profound in patients with pre-existing conditions. For example, García-Suárez et al. studied the effects of COVID-19 on cancer patients and found that those who contracted COVID-19 had a 50% higher likelihood of cancer progression compared to those who did not. This study highlighted a significant interaction between COVID-19 and pre-existing cancer, suggesting that the virus could exacerbate cancer progression through immune modulation [13]. These findings suggest that the virus not only promotes cancer development but also complicates its management, potentially leading to worse prognoses for cancer patients who contract COVID-19. A study by Mehta et al. found that approximately 20% of severe COVID-19 patients experienced a cytokine storm, leading to elevated levels of inflammatory markers such as IL-6, C-reactive protein, and ferritin [14]. These markers were significantly higher in patients who required ICU admission compared to those with milder symptoms (p < 0.001). Research by Coperchini et al. highlights that chronic inflammation resulting from COVID-19 can persist even after recovery from the acute phase. In a cohort of recovered patients, elevated inflammatory markers were observed for up to three months post infection [15]. In a study by Neves et al., this prolonged inflammatory state is associated with oxidative stress and DNA damage, as evidenced by increased levels of 8-hydroxy-2’-deoxyguanosine (8-OHdG) (p < 0.05) [16]. Furthermore, a meta-analysis by Wu et al. involving over 15,000 COVID-19 patients from multiple studies revealed a significant association between high levels of inflammatory cytokines and increased rates of cancer-related gene expression [17]. The analysis showed that patients with severe COVID-19 had a 1.5-fold increase in the expression of genes involved in cell proliferation and survival pathways (p < 0.01).

It has been reported that excessive proinflammatory cytokine release, commonly referred to as a cytokine storm, during a severe COVID-19 infection can lead to chronic inflammation and tissue damage. This state of chronic low-grade inflammation, particularly in “long COVID” syndrome, is associated with increased levels of cytokines, growth factors, and chemokines in the plasma. Rahimmanesh et al. in their review, highlight several mechanisms through which severe COVID-19 can increase cancer risk. They note that severe COVID-19 patients exhibit extremely high levels of proinflammatory cytokines like IL-6 and TNF-α. Additionally, persistent inflammation in “long COVID” syndrome includes elevated cytokines and chemokines, which can promote tumor growth. The hypoxic microenvironment due to inflammation increases the production of reactive oxygen species and lysyl oxidase, facilitating tumor cell invasion and metastasis. The study also notes the activation of oncogenic pathways such as JAK-STAT, mitogen-activating protein kinase (MAPK), and NF-κB, which can increase cancer risk. These inflammatory responses can potentially facilitate cancer progression and recurrence by promoting oxidative stress, DNA damage, and a hypoxic microenvironment that favors tumor cell invasion and metastasis [18].

A study by Andrejkovits et al. involved 95 COVID-19 patients categorized into mild (n = 29), moderate (n = 29), and severe/critical (n = 37) groups. The study found a significant increase in IL-10 and IFN-γ levels in severe/critical cases compared to mild cases (p = 0.044 and p = 0.025, respectively). It also showed significant decreases in total thiol, native thiol, and disulfide levels in severe/critical cases (p = 0.004, p = 0.006, and p = 0.003, respectively), indicating increased oxidative stress. Furthermore, the percentage of tail DNA, an indicator of DNA damage, was significantly higher in severe/critical cases compared to mild cases (p = 0.022). In severe cases of COVID-19, markers such as IL-10 and IFN-γ were elevated, and there was a notable increase in DNA damage indicators, suggesting that the prolonged inflammatory response in severe COVID-19 can contribute to genotoxic stress, which is a known precursor to carcinogenesis [19].

These findings underscore the complex interplay between chronic inflammation caused by COVID-19 and the potential for increased cancer risk. Table 1 describes the basic mechanism of how carcinogenesis is caused by COVID-19.

**Table 1:** Basic mechanisms of COVID-19-related carcinogenesis

Mechanism	Description
Immune dysregulation	COVID-19 causes significant immune responses, including cytokine storms, leading to chronic inflammation, a known risk factor for cancer development [6-10].
Chronic inflammation	Persistent inflammatory responses may result in DNA damage, promote cellular proliferation, and inhibit apoptosis, thereby facilitating carcinogenesis [6-10].
Oxidative stress	Elevated oxidative stress levels in COVID-19 survivors are linked to an increased risk of cancer through DNA damage and promotion of a tumor-friendly environment [12,18,19].
Genetic and epigenetic changes	SARS-Cov-2 influences gene expression through DNA methylation and histone modification, impacting oncogene and tumor suppressor gene expression [20-24].

Genetic and epigenetic changes associated with COVID-19

The impact of COVID-19 on genetic and epigenetic changes has garnered significant attention in the scientific community, particularly in the context of carcinogenesis. Alterations in DNA methylation patterns have been observed in COVID-19 patients, potentially leading to the silencing of tumor suppressor genes. This perspective explores the findings related to the genetic and epigenetic alterations associated with SARS-CoV-2 infection, as well as their potential implications for cancer development (Table 1).

COVID-19 has been linked to various genetic and epigenetic changes that may contribute to carcinogenesis. One of the primary mechanisms through which SARS-CoV-2 influences gene expression is by modulating DNA methylation and histone modification. These alterations can impact the expression of oncogenes and tumor suppressor genes, thereby influencing the cellular processes that lead to malignant transformation.

DNA methylation is a critical epigenetic modification that plays a role in regulating gene expression. The hypermethylation of tumor suppressor genes is a well-documented pathway through which viruses can contribute to oncogenesis. In COVID-19 patients, alterations in DNA methylation patterns have been observed, particularly hypermethylation of tumor suppressor genes. These findings align with other studies on viral infections and epigenetic changes. For instance, Epstein-Barr virus (EBV) and human papillomavirus (HPV) have been shown to induce similar DNA methylation changes that can lead to cancer development [20,21]. In a cohort conducted by Balnis et al., DNA methylation profiles were determined using data from 128 patients with (n = 102) and without (n = 26) [22]. COVID-19 diagnoses were collected simultaneously. This study reported that there was no significant difference between the groups in terms of average DNA methylation across the entire genome (COVID-19 patients: 58.5%; non-COVID-19 patients: 58.4%). On the other hand, a linear regression model was created to investigate locus-specific DNA methylation levels associated with SARS-CoV-2 infection. As a result, it was stated that COVID-19 patients showed changes at specific DNA positions even when compared to patients with acute respiratory decompensation due to other causes [22].

Histone modifications, including acetylation and methylation, are essential for regulating gene expression. In a study conducted by Huckriede et al. on 117 COVID-19-positive patients, increased histone H3 levels were observed during admission to the ICU, with 50% of patients reported to have detectable histone H3 levels at least once during their ICU stay. This study stated that any histone H3 detection may be associated with an increase in thromboembolic events and secondary infections [23]. These observations are consistent with previous research on the role of histone modifications in COVID-19. Histone acetylation is known to be involved in gene activation, and its dysregulation has been implicated in various cancers. Histone methylation, on the other hand, can either activate or repress gene expression depending on the specific histone mark, and its alteration is a common feature in cancer cells [24].

The mechanisms underlying these genetic and epigenetic changes in COVID-19 patients involve direct viral effects, immune responses, and inflammation. SARS-CoV-2 can modulate host cellular machinery, leading to changes in DNA methylation and histone modifications. Additionally, the inflammatory response and cytokine storm associated with COVID-19 may further contribute to these alterations [18,19]. In a longitudinal prospective cohort study by Nikkhoo et al., 208 confirmed COVID-19 patients were divided into two groups according to their IL-6 values ​​on the first day of hospitalization: high (n = 107) or non-high/normal (n = 101). Although not significant, logistic regression results showed that patients with high IL-6 had a 3.91-fold higher probability of death. However, IL-6-mediated cytokine storm is considered an important indicator of inflammation [25]. The role of inflammation in cancer has been well-established, with chronic inflammation being a risk factor for various types of cancer. The cytokine storm observed in severe COVID-19 cases can create a pro-inflammatory environment that may facilitate these epigenetic changes, similar to what has been observed in other inflammatory conditions [26].

These findings highlight the potential long-term impact of COVID-19 on cancer risk. In conclusion, the modulation of oncogenes and tumor suppressor genes through epigenetic changes can promote malignant transformation and tumor development (Table 1). Understanding these mechanisms is crucial for developing targeted interventions to mitigate the cancer risk associated with COVID-19.

Basis of virological and molecular properties of CoVs

CoVs have been reported to be a large family of enveloped, spherical, single-stranded, positive-sense RNA viruses belonging to the *Nidovirales* order and have a major surface protein called spike (S) protein [27-29]. CoVs infect a wide variety of mammalian and avian species, causing respiratory or enteric diseases, and this infection begins by binding of the S protein to the receptor and fusion of the viral lipid envelope with cellular membranes [30,31].

Like fusion proteins, which are important for many other viruses to play a role as pathogens, S protein, which is the fusion protein of CoVs, is activated by cellular proteases [31-33]. Activation of the S proteins of the CoVs requires a rather complex process, and this activation results in the production of S1 and S2 subunits that are non-covalently linked to the proteolytic cleavage of the S protein in two different parts, defined as S1/S2 and S2'. As a result of molecular studies, it was stated that the S1 subunit contains a receptor binding domain and the S2 subunit contains the fusion machine connected to the membrane [30,32,34]. It is stated that cleavage in the S2 portion just above the hydrophobic fusion peptide triggers the membrane fusion activity of the S protein. In contrast, the importance of S protein cleavage in the S1/S2 domain is unclear. Nevertheless, based on the information obtained after molecular controls, it is accepted that the S protein in CoVs occurs in a successive complex process with cleavage in the S1/S2 region and cleavage in S2, respectively [35-39].

It has been stated that cleavage in the S1/S2 domain, within this complex process, can be very important in terms of conformational changes required for the binding of the S protein to the receptor and/or the S2' site appearing consecutively to be exposed to the proteases at the stage of entry into the host cell [34-36]. Proteases involved in this phase include FURIN, cathepsin-L, and trypsin-like serine proteases such as transmembrane serine protease (TMPRSS) 2, TMPRSS11A, and TMPRSS11D, and these proteases have been shown to activate CoVs in vitro. Among these listed proteases, TMPRSS2 and FURIN have been found to play an important role in proteolytic activation of various viruses [34-37].

The physiological role of TMPRSS2, commonly expressed in epithelial cells of the respiratory, gastrointestinal, and urogenital systems, is not yet clear [38-41]. However, more recent studies have shown that TMPRSS2 can activate fusion proteins of a number of respiratory viruses including human metapneumovirus and human parainfluenza viruses, as well as CoVs including SARS-CoV and the MERS-CoV in vitro [39,40]. As a result of the studies, it was shown that mice with TMPRSS2 deficiency did not suffer from serious pathogenic results when infected with influenza A virus strains, SARS-CoV and MERS-CoV [38-40]. This situation is tried to be explained with two hypotheses: the first is the inhibition of the proteolytic activation of the progeny virus, and consequently, the second is the inhibition of the virus spreading along the respiratory tract [4,32,39]. Along with the data obtained as a result of these studies, the use of virus-activating host cell proteases, especially TMPRSS2 inhibition, was the goal in the treatment of respiratory virus infections [38-40].

FURIN is a member of the secretory proprotein convertase (PC) family consisting of PC1/3, PC2, FURIN, PC4, PC5/6, PACE4, PC7, SKI-1/S1P, and PCSK9 [41]. PCs are responsible for the activation of a wide variety of precursor proteins such as growth factors, hormones, receptors, adhesion molecules, and cell surface glycoprotein of infectious viruses; proteolysis plays a role in this activation [32,41]. It is considered a type I transmembrane protein that is expressed in eukaryotic tissues and cells. FURIN isolates precursors of a wide variety of proteins, including hormones, growth factors, cell surface receptors, and adhesion molecules, during transport along the secretion path, and is considered to be performed in R-X-R/K-R↓ multibasic motif sequences. FURIN is considered an activating protease for fusion proteins of a wide variety of viruses such as highly pathogenic avian influenza A virus (HPAIV), HIV, Ebola virus, Measles virus, and Yellow Fever virus. Similarly, multi-base motifs include bacterial toxins such as Shiga toxin or anthrax toxin. Most of the time, the infection spreads systemically and fatally as a result of activation of HA, the surface glycoprotein of HPAIV, with FURIN. In contrast, the HA cleavage site of low pathogenic avian influenza A virus (LPAIV) is activated in a monobasic cleavage site by means of serine trypsin-like serine proteases. It is thought that this division is expressed in the respiratory and intestinal tract of birds and limits the spread of infection to these tissues [41].

Based on the previous information, it has been concluded that proteases that are properly expressed in the respiratory and intestinal tract of birds limit the spread of infection to these tissues [39]. Recent data have shown that TMPRSS2 also plays a role in the activation of the S protein of SARS-CoV-2. Cell line studies have shown that temporary expression of TMPRSS2 in Vero cells supports the entry of cathepsin-independent SARS-CoV-2 pseudotypes. Current data suggest that the sequence analysis of the S protein of SARS-CoV-2 may also be involved in FURIN S processing. The S1/S2 region of the SARS-CoV-2 S protein has been shown to include the addition of four amino acids that provide a minimum FURIN cleavage site (R-R-A-R685↓), unlike the S protein of SARS-CoV. Instead, similar to SARS-CoV, S2' cleavage domain of the SARS-CoV-2 S contains a matched dibasic motif with 134 single KR segments (KR815↓) recognized by trypsin-like serine proteases. This may explain the difference in the pathogenic effect of SARS-CoV-2 [41,42].

Spikes on the surface of the viral particle together with the spherical morphology look like a “crown” in electron microscopy, and therefore these viruses are called corona, which means royal crown in Latin. CoVs are envelopes with an envelope consisting of a lipid bilayer derived from the host cell membrane. Its primary viral structure is proteins other than S protein, such as membrane (M), envelope (E), and nucleocapsin (N). In some beta-coronaviruses, hemagglutinin esterase (HE) protein is also included in this primary viral structure. SARS-CoV-2 is also classified as a beta-coronaviruses [41].

The spike glycoprotein in the nCoV has been found to contain a FURIN-like cleavage site that is not in the CoV of the same clan. The S protein of CoVs is responsible for receptor binding, tissue tropism, and pathogenesis. The activation of the trimetric S protein by host cell proteases during the infection process takes place at the S1/S2 cleavage site of this protein. Following splitting, also known as the preparation stage of activation, the S protein is divided into two: (i) N-terminal S1-ectodomain recognizing the cognate cell surface receptor and (ii) a C-terminal S2-membrane-anchored protein involved in viral entry [1,39,41,42]. 

The SARS-CoV S1-protein contains a protected receptor binding domain (RBD) that recognizes ACE2. SARS-CoV is known to bind to both bat and human cells, and the virus infects both organisms. It is thought that there is a similar S1 protein-ACE2 receptor interaction, even if the entire mechanism for SARS-CoV-2 has not been clearly demonstrated [1,4,39,41,42].

Possible proteins of SARS-CoV that may be associated with carcinogenesis

Given the established relationship between COVID-19 and cancer risk through mechanisms of immune dysregulation, cytokine storms, and genetic and epigenetic changes, it is essential to explore specific molecular pathways and proteins that mediate these effects. Integrating these findings with the hypothetical roles of ACE2, TMPRSS2, and FURIN in cancer development provides a more comprehensive understanding of the potential mechanisms at play.

*ACE2 Receptor and Carcinogenesis * 

The role of the ACE2 receptor in CoV-2 infection: ACE2 is a newly detected component of the renin-angiotensin-aldosterone system (RAAS) and has been shown to be moderately expressed in the lungs of both humans and mice. ACE2 expression was also detected in the kidney, heart, testicle, and small intestine cells of humans and mice. ACE2 functions in two different pathways in its RAAS. The first is to catalyze the conversion of AGTI to AGT (1-9), and the second is to catalyze the conversion of AGTII to AGT (1-7), which is a peptide. Although the vasodilation and anti-proliferative properties of AGT (1-7) are known, AGT (1-9) has no physiological significance [1,4,43-51].

Previous experience has comprehensively explained the process of receptor recognition by SARS-CoV. The S protein located on the surface of the SARS-CoV binds to the ACE2 receptor through the RBD, and as a result of this interaction, SARS-CoV enters the host cell. The RBD, which was found to be specific to the ACE2 receptor, was determined to contain a core and a receptor binding motif (RBM). The RBM has been reported to mediate contact with ACE2, thereby regulating the infectivity, pathogenic features, cross-species diversity as well as human-to-human transmissions of the SARS-CoV [1,4,43-51].

The sequence similarity between the S proteins of SARS-CoV and SARS-CoV-2 has gradually increased the views that SARS-CoV-2 uses ACE2 as the receptor. Some studies have even shown that SARS-CoV2 is more selective than SARS-CoV in terms of affinity to ACE [42-51]. However, it can also be said that the mechanism for the interaction between the S protein of SARS-CoV-2 and the ACE2 receptor is not yet clear. After interacting with SARS-S protein and ACE2, cellular TMPRSS2 is activated for the production of S protein in the host cell [39,41,42,52,53]. However, the role of TMPRSS2 in the host cell entry of the SARS-2-S is not yet clear. Nevertheless, since SARS-CoV-2 is similar in terms of cell entry mechanism to SARS-CoV, the idea that what is known about SARS-CoV may also apply to SARS-CoV-2 [42-53]. Therefore, the binding affinity of S protein and ACE2 is thought to be closely related to the rate of replication and disease severity for SARS-Cov-2, such as SARS-CoV. Additionally, it is believed that cathepsin B/L activity at the cell entry can replace TMPRSS2 [52,53].

It has recently been discovered that a path different from the conventional RAAS plays an important role. This new path includes ACE2 and AGT (1-7) [53-56]. ACE2 converts AGTII to AGT (1-7), and AGT (1-7) interacts with its specific MAS receptor, performing the opposite of AGTII's functions [53-56]. While current data have shown a significant relationship between serum AGTII levels and viral load in patients with COVID-19, no clear data on AGT1-7 levels are available yet. Neutrophil accumulation, microvascular thrombosis, and fibrosis induction functions of AGTII are inhibited by balancing it with AGT (1-7) [56]. Implications from clinical observations may suggest that the modulating effect of this equilibrium state may be impaired in severe COVID-19 patients. This may hypothetically suggest that the AGTII pathway is overactive and the overexpression of these pathway-related peptides in patients with COVID-19 [52-56].

It has been reported that the gene expression level of ACE2 may be associated with susceptibility to SARS-CoV-2 infection, and TMPRSS22 plays a supportive role. It was also emphasized that TMPRSS2 is severely downregulated in lung cancer tissues and therefore may be a tumor suppressor gene [1,42,50,52,56].

The possible relationship of ACE2 with carcinogenesis: Immunohistochemical studies have shown that ACE2 and TMPRSS2 are extensively expressed in both nasal and bronchial epithelium. In studies related to SARS-CoV, it has been stated that ACE2 and TMPRSS2 gene expression mostly occurs in alveolar type II epithelial cells but there is no expression in the upper airways [1,4,57-58]. However, in studies for SARS-CoV-2, there are important findings that ACE2 and TMPRSS2 gene expression can be expressed in many tissues in the body, although the results may not be clear [6,34-37]. Studies have demonstrated that severe COVID-19 patients exhibit extremely high levels of proinflammatory cytokines like IL-6 and TNF-α, which contribute to chronic inflammation and oxidative stress. This environment can promote tumor growth through hypoxia-induced oxidative stress and activation of oncogenic pathways such as Janus kinase/signal transducers and activators of transcription (JAK/STAT), mitogen-activated protein kinases (MAPKs), and nuclear factor kappa-light-chain-enhancer of activated B cells (NF-κB) [18,19].

AGTII, the effective major peptide of the RAAS, known to play a role in different physiological and pathological processes, is produced by ACE from AGTI. This peptide also acts proangiogenically after binding to AGTII receptor-1 (ATGIIR1) and has therefore been associated with tumorigenesis [58-61]. Although there are different results regarding carcinogenesis and its role in cancer patients, ATGIIR1 and ACE expression are thought to be associated with poor prognosis in many cancers. The downregulation of ACE2 due to SARS-CoV-2 infection can lead to increased AGTII levels, promoting angiogenesis and tumor growth through ATGIIR1 activation [58,60-62].

While ATGIIR1 overexpression has been associated with invasion and angiogenesis in ovarian cancer, the insertion/deletion polymorphism (ACE I/D) of ACE, especially the DD-genotype, is involved in early gastric cancer development and increased lymph node metastasis. Excessive ATGIIR1 expression and ACE I/D polymorphism have been reported to be independent risk factors for nodal metastasis in intestinal gastric cancer. In animal studies of head and neck cancers and colorectal carcinoma, ATGIIR1 and ACE inhibition have been found to stop tumor proliferation [60-66]. Conversely, activation of the ACE2/AGT1-7/Mas receptor pathway has been shown to inhibit proliferation in lung and colon cancer cell lines due to AGT1-7 acting antagonistically to AGTII [58,60-62].

Activation of many intracellular signaling pathways is thought to play a role in the effect of AGTII on tumoral proliferation. Although not clear, activation of AGTII can affect tumor development by activating the ERK and JNK pathways of the MAPK pathway. Similarly, there may be a relationship between AGTII activation and vascular endothelial growth factor. However, there is evidence that the p38MAPK pathway in the MAPK pathway works in the opposite direction with ACEII/AGT (1-7)/MAS receptor activation [58,60-62].

As a result, COVID-19, which is encountered with very different clinical presentations caused by SARS-CoV-2, may decrease ACE2 levels due to endocytosis with the virus [1,42,52-59]. However, in hosts where it does not cause death by cytokine storm caused by the excessive increase of AGTII, AGTII may perhaps initiate carcinogenesis with the effect of viral genomic integration [1].

These findings illustrate how the severe inflammatory response and prolonged oxidative stress in COVID-19 patients can contribute to an environment that potentially supports carcinogenesis. The downregulation of ACE2 due to SARS-CoV-2 infection exacerbates these effects by increasing AGTII levels and promoting oncogenic pathways.

TMPRSS2 and Carcinogenesis

TMPRSS2 is a pericellular protease. These enzymes that degrade extracellular matrix proteins have been found to play a role in cancer cells crossing the basement membrane and spreading to surrounding tissues. The relationship between TMPRSS2 and carcinogenesis has been clearly defined in prostate cancer and it has been emphasized that it plays an important role in androgen signaling [53]. However, there is no clear data on the relationship of TMPRSS2 to other cancer types. Similarly, no data was reported on an interaction between TMPRSS2 and another member of the same family, TMPRSS4, in carcinogenesis [53,54]. However, TMPRSS4, which was first described in pancreatic cancer, has been reported to be overexpressed in ovarian, thyroid, colorectal, breast, cervix, gallbladder, stomach, and liver cancers [53-55]. There is a study on the pathogenesis of COVID-19 that reports that there is an interaction between TMPRSS2 and TMPRSS4 in small intestinal epithelial cells [54]. This may suggest the presence of crosstalk or an unknown carcinogenic pathway between these two molecules that are members of the same family.

TMPRSS2’s role in degrading extracellular matrix proteins facilitates cancer cell invasion and metastasis. Its clear role in prostate cancer through androgen signaling suggests that similar pathways might be involved in other cancers. Although there is limited data on TMPRSS2’s role in cancers other than prostate cancer, its interaction with TMPRSS4, known to be overexpressed in several types of cancers, indicates a potential broader role in carcinogenesis. The interaction between TMPRSS2 and TMPRSS4 in the context of COVID-19 suggests a synergistic effect on cancer development, possibly through enhancing the ability of cancer cells to invade and metastasize [18].

FURIN and Carcinogenesis

FURIN, a calcium-dependent protease expressed throughout the body in all vertebrates and many invertebrates, has also been shown to be expressed in cancer cells. In various types of cancer, FURIN activity has been reported to promote many cancer-related processes such as cell proliferation, migration and invasion, or vascularization [67-70]. FURIN, a proprotein convertase that is distributed everywhere in the tissue, has been shown to be overexpressed in lung, head and neck, colon, gynecological cancers, and sarcomas [67-70]. There are even conclusions that FURIN is a driver of pro-oncogenes in *KRAS* and *BRAF* mutant colorectal cancer [68]. The studies highlight the role of hypoxia and oxidative stress in promoting tumor growth. Proinflammatory cytokines, chronic inflammation, and activation of oncogenic pathways are central to this process [18,19].

The widespread expression of FURIN and its role in promoting cancer-associated processes such as cell proliferation, migration, and invasion are consistent with the effects of chronic inflammation and oxidative stress observed in COVID-19 patients. However, there are no clear study results yet on the relationship of FURIN to carcinogenesis in COVID-19.

Table 2 shows the role and mechanism of some viral proteins in triggering carcinogenesis.

**Table 2:** Potential viral proteins triggering carcinogenesis ACE2: angiotensin-converting enzyme 2; TMPRSS2: transmembrane serine protease 2; ATGIIR1: angiotensin II receptor-1

Protein/ Molecule	Role in Carcinogenesis	Key Mechanism	Relevant Cancer Types
ACE2 [65-68]	Downregulation due to SARS-COV-2 infection may increase AGTII levels, promoting tumor growth via the ATGIIR1 pathway.	Facilities tumorigenesis by promoting angiogenesis and cellular proliferation	Lung, kidney, testicle, and small intestine cancers
TMPRSS2 [54]	Degrades extracellular matrix proteins, facilitating cancer cell invasion and metastasis. Overexpressed in COVID-19 cases.	Supports viral entry and possibly enhances cancer cell invasiveness	Prostate cancer, potential in other cancers through interaction with TMPRSS2
FURIN [69,70]	Promotes cell proliferation, migration, invasion, and vascularization in various cancers, overexpressed in COVID-19 cases.	Drives oncogenic processes, especially in *KRAS* and *BRAF* mutant colorectal cancer	Lung, head and neck, colon, gynecological cancers, sarcomas

COVID-19 therapeutics and carcinogenic potential

The use of therapeutic agents such as corticosteroids and immunosuppressive drugs in treating COVID-19 has been instrumental in managing the disease’s severe manifestations. However, these treatments come with potential long-term carcinogenic risks. Long-term use of these medications can impair immune surveillance and promote oncogenic viral infections, thus increasing the risk of cancer development [6-10].

While corticosteroids are effective in reducing inflammation and managing severe COVID-19 symptoms, their long-term use can suppress the immune system, making the body more susceptible to infections, including those caused by oncogenic viruses like EBV and HPV [71]. Bahsoun et al.'s study documented the immunosuppressive effects of corticosteroids, which can dampen the body’s natural ability to detect and eliminate pre-cancerous cells, thereby potentially increasing cancer risk [72].

Immunosuppressive drugs are often used to prevent severe immune reactions in COVID-19 patients. However, their immunosuppressive nature can impair the body’s ability to detect and destroy cancerous cells. A study by Mehta et al. declared that COVID-19 patients treated with immunosuppressive drugs had a higher incidence of subsequent infections, which could include oncogenic viruses [14]. The long-term use of these drugs can disrupt the delicate balance of immune surveillance, thereby potentially facilitating the development of cancer.

The therapeutic strategies employed to manage COVID-19, while effective in controlling the acute phase of the disease, may have long-term consequences that include an increased risk of cancer. The use of corticosteroids and immunosuppressive drugs, essential in managing severe COVID-19 cases, must be carefully balanced against their potential carcinogenic effects.

Co-infections and cancer risk

The interplay between co-infections of EBV and HPV with COVID-19 has drawn considerable attention in recent literature due to its profound implications on immune dysregulation and cancer development.

Previous studies have shown that co-infections can lead to more severe disease outcomes due to compounded immune modulation and inflammatory responses [73]. These results underscore the significant role of viral co-infections in enhancing the oncogenic landscape in COVID-19 patients, corroborating earlier findings on the multiplicative effects of co-infections on immune dysregulation.

The evidence strongly suggests that co-infections with EBV and HPV in COVID-19 patients lead to significant increases in inflammatory markers and tumorigenic potential. This highlights the critical need for comprehensive monitoring and management of co-infections in COVID-19 patients to mitigate the enhanced risk of cancer development [73-75]. In their study, Bernal and Whitehurst collected 106 blood plasma samples from COVID-19-positive and negative patients [74]. Based on quantitative polymerase chain reaction (qPCR) detection of EBV genomes, they detected EBV reactivations in 27.1% (13/48) in the COVID-19-positive group, while this rate was only 12.5% ​​(6/48) in the COVID PCR-negative group. However, they found that 42.30% (20/52) of the COVID PCR-negative group had detectable antibodies against the SARS-CoV-2 nucleoprotein, indicating past infection.

Interestingly, a decrease in the frequency of HPV infection has been shown as a possible result of social isolation experienced during the pandemic. In one of the studies reviewed, Yi et al. analyzed a total of 44,401 genital swab samples taken in two separate periods, covering the pre- and post-pandemic periods, and showed that the overall HPV infection rate decreased from 33.43% (11,245/33,531) to 29.43% (5,527/18,780) during the pandemic period compared to the pre-pandemic period [75]. Nevertheless, it is thought that COVID-19 and HPV coinfection may hypothetically be a contributor to triggering carcinogenesis, but no clear conclusion can be reached on this matter.

The synergistic effects of these oncogenic viruses underscore the complexity of the immune response in the context of COVID-19 and emphasize the importance of addressing these co-infections in clinical settings.

Synchronous and metachronous second primary cancers

Synchronous second cancers are defined as two or more primary cancers diagnosed simultaneously or within a six-month period. COVID-19’s impact on the immune system may increase the likelihood of developing synchronous second cancers due to immune suppression and chronic inflammation. These cases suggest that the virus may enhance oncogenic processes through chronic inflammation and immune suppression, creating an environment conducive to the simultaneous development of multiple primary tumors. Chronic inflammation is a well-known risk factor for cancer, as it can lead to DNA damage, promote cellular proliferation, and create a pro-tumorigenic microenvironment. The persistent inflammatory state post-COVID-19 recovery might contribute to an environment where multiple tumors can develop simultaneously. Studies investigating the effects of COVID-19 on the immune system have hypothesized that the cytokine storm and chronic inflammation caused by the virus may trigger cancer development. Characterized by an excessive immune response, the cytokine storm can cause tissue damage and create an environment conducive to tumor formation [76,77].

Pinato et al. conducted a study on the long-term health outcomes of COVID-19 survivors and found that those with severe COVID-19 were more likely to develop cancers within two years post-recovery [78]. They proposed that prolonged immune dysregulation and chronic inflammation post-infection could be key drivers of this increased cancer risk. Although there is no clear evidence that COVID-19 may lead to an increase in the frequency of leukemia and lymphoma, this hypothesis has been tried to be elucidated with case series [77]. Indeed, it is thought that exaggerated activation of the classical arm of the RAS (i.e. ACE/ANGIIAT1R) may promote neoplastic hematopoiesis through various mechanisms. This has been linked to the two-hit hypothesis, which leads to an expanded leukocyte clone circulating in the peripheral blood, now referred to as clonal hematopoiesis of uncertain potential. On the other hand, two other important views proposed to explain the hypothesis that there may be an increase in the frequency of both leukemia and lymphoma after COVID-19 are: the effect of the inflammatory role of increased cytokines in COVID-19 and the coincidence situation [77].

Changes in cancer behavior post COVID-19

Emerging data suggest that COVID-19 may have influenced the behavior and molecular characteristics of certain cancers, including breast cancer. Studies reported that breast cancer patients diagnosed post-COVID-19 showed more aggressive tumor behavior compared to those diagnosed pre-COVID-19 [78,79]. This includes higher proliferation rates and an increased incidence of triple-negative breast cancer, which is typically more difficult to treat and has a poorer prognosis. In a retrospective study by Negrao et al., two periods were analyzed: March-October 2019 (pre-COVID period) and March-October 2020 (COVID period). In comparing the data of 115 women newly diagnosed before the pandemic with 59 women diagnosed during the COVID period, the frequency of triple-negative tumors was twice as high (10.1% vs. 21.6%, p = 0.062) [79].

Table 3 describes the features of post-COVID-19 cancer development.

**Table 3:** Features of cancer development post-COVID-19 IL-6: interleukin-6; TNF-α: tumor necrosis factor alpha; JAK-STAT: Janus kinase/signal transducers and activators of transcription; MAPK: mitogen-activated protein kinase; NF-κB: nuclear factor kappa-light-chain-enhancer of activated B cells

Cancer Development Feature	Details	Key Findings
Increased cancer risk	COVID-19 survivors show an increased risk of cancer development, particularly due to immune dysregulation and chronic inflammation.	Increased IL-6 and TNF-α levels, oxidative stress markers, prolonged inflammation [18].
Epigenetic alterations	Alterations in DNA methylation patterns, particularly hypermethylation of tumor suppressor genes, contributing to malignant transformation.	Hyperactive oncogenic pathways (e.g. JAK-STAT, MAPK, NF-κB) identified in post-COVID cases [18,19].
Synchronous and metachronous cancers	Post-COVID-19, there is an increased occurrence of synchronous and metachronous primary cancers, possibly due to immune suppression and chronic inflammation.	Studies show higher incidence of multiple primary tumors and hematological malignancies such as lymphoma and leukemia [76-78].
Rare cell type cancers	Increased incidence of rare cancers such as small cell carcinoma and angiosarcoma, potentially linked to COVID-19-induced immune dysregulation.	Significant rise in rare cell type cancers documented in post-COVID-19 cases [80-82].

Increase in rare cell type cancers

There is also evidence suggesting an increase in the incidence of rare cell-type cancers post-COVID-19 (Table 3). For instance, several studies documented a rise in the diagnosis of rare cell-type cancers such as small cell carcinoma and angiosarcoma in the post-COVID-19 period [80-82]. The mechanisms behind this increase are not fully understood but may relate to COVID-19-induced immune dysregulation and chronic inflammation promoting oncogenesis in these rare cell types. This finding emphasizes the importance of ongoing monitoring to understand the long-term cancer risks associated with COVID-19. Continuous surveillance of COVID-19 survivors is crucial to detect and manage secondary cancers early.

Future research directions

Future research should focus on longitudinal studies to monitor cancer incidence in COVID-19 survivors over extended periods. Investigating the molecular mechanisms underlying SARS-CoV-2-induced genetic and epigenetic changes will also be crucial. Additionally, evaluating the long-term safety of COVID-19 therapeutics with respect to cancer risk is necessary.

## Conclusions

The relationship between COVID-19 and cancer development is a complex interaction of immune suppression, chronic inflammation, genetic and epigenetic changes, and possible direct viral effects. In particular, viral proteins such as ACE2, FURIN, and TMPRSS2 are highly likely to be associated with cancer progression. However, the effect of these proteins on carcinogenesis after COVID-19 is not yet clear. The increasing frequency of both synchronous and metachronous second primary cancers in COVID-19 survivors requires greater awareness and long-term monitoring.

All these findings emphasize the importance of integrating cancer surveillance into the post-recovery care of COVID-19 patients and warrant further research to elucidate the underlying biological mechanisms. In addition, when all these studies are evaluated together with their results at the molecular level, it may be thought that COVID-19 may initiate carcinogenesis or worsen existing cancer through immune suppression and chronic inflammation, as well as genetic and epigenetic changes it may cause. Detailed studies on specific proteins and ongoing longitudinal studies will inform efforts to develop new agents for both cancer prevention and cancer treatment.
